# Genetic code degeneracy is established by the decoding center of the ribosome

**DOI:** 10.1093/nar/gkac171

**Published:** 2022-03-24

**Authors:** Shixin Ye, Jean Lehmann

**Affiliations:** INSERM U1195 unit, University of Paris-Saclay, 94276 Le Kremlin Bicêtre, France; Institute for Integrative Biology of the Cell (I2BC), CEA, CNRS, University of Paris-Saclay, 91198 Gif-sur-Yvette, France

## Abstract

The degeneracy of the genetic code confers a wide array of properties to coding sequences. Yet, its origin is still unclear. A structural analysis has shown that the stability of the Watson–Crick base pair at the second position of the anticodon–codon interaction is a critical parameter controlling the extent of non-specific pairings accepted at the third position by the ribosome, a flexibility at the root of degeneracy. Based on recent cryo-EM analyses, the present work shows that residue A1493 of the decoding center provides a significant contribution to the stability of this base pair, revealing that the ribosome is directly involved in the establishment of degeneracy. Building on existing evolutionary models, we show the evidence that the early appearance of A1493 and A1492 established the basis of degeneracy when an elementary kinetic scheme of translation was prevailing. Logical considerations on the expansion of this kinetic scheme indicate that the acquisition of the peptidyl transferase center was the next major evolutionary step, while the induced-fit mechanism, that enables a sharp selection of the tRNAs, necessarily arose later when G530 was acquired by the decoding center.

## INTRODUCTION

The translation of genetic information relies on base pairing between anticodons and codons. While the first two codon positions are restricted to canonical Watson–Crick base pairs, some flexibility occurs at the third position. This flexibility was postulated by F. Crick in 1966 to account for the observed degeneracy in the genetic code, which had just been fully deciphered (1). He suggested that G could base pair not only with C but also with U if some base displacement was possible at the third position, a possibility coined the ‘wobble hypothesis’ (2). This flexibility would allow reduced sets of tRNAs to translate all amino-acid encoding codons, thereby making translation more efficient. The reason why unspecific pairing can be accepted at the third position became apparent only about 35 years later when the first structures at atomic resolution of the 30S subunit co-crystallized with mRNA fragments and anticodon stem-loops were elucidated (3). These structures revealed that, unlike at the first and second position, the ribosome does not structurally constrains the wobble position, implying that some flexibility in the geometry of base pairing is possible (3–5).

In the meantime, it was discovered that extended wobbling, called ‘superwobbling’ (6), can also occur at the third position. In that case, an unmodified U (exceptionally an A) at position 34 of a tRNA can base pair with any base at the third position of the codons (7). So far, superwobbling has been observed only in mitochondria, chloroplasts and other small genome entities with reduced sets of tRNAs (7–9). In such cases, the extent of wobbling matches the degeneracy families associated with each of the 16 N_1_N_2_ codon doublets of the genetic code: all codons of any codon family, whether it is two- or four-fold degenerate, are translated by a single tRNA through wobbling and superwobbling, respectively.

**Figure 1.**
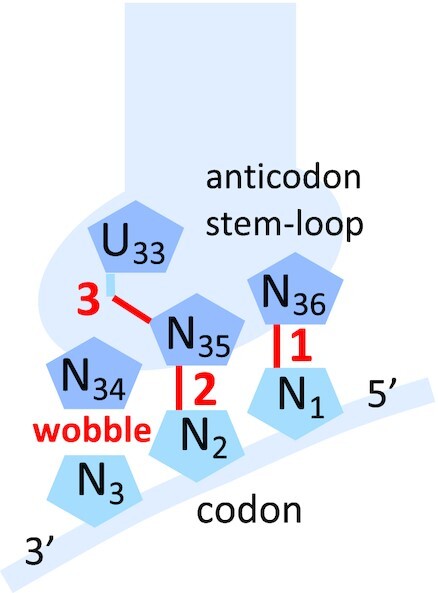
Anticodon–codon interaction and structural interpretation of the three parameters of Lagerkvist (in red). See text for explanations.

The rationale behind the existence of these two degeneracy families was partially unraveled in 1978 by U. Lagerkvist, who noticed that the strength of the base pairs in positions 1 and 2 of the codons and the purine/pyrimidine nature of the base at the second position constituted a set of three criteria (or parameters) with which a complete categorization of the 16 codon doublets into the two degeneracy families was possible (10), a feature that can be highlighted by a symmetry in the genetic code table (11). Based on the then available structural organization of the decoding center, and the architecture of the anticodon loop, an interpretation of these parameters was proposed in 2008. The analysis (12) demonstrated that all three parameters of Lagerkvist determine the number of hydrogen bonds contributing to the stability of the WC geometry of the base pair at the second position of the anticodon (N_35_-N_2_), as follow (Figure 1):

- Parameter 1: number of hydrogen bonds established by the WC base pair at the first codon position (N_36_-N_1_).
- Parameter 2: number of hydrogen bonds established by the second base pair itself (N_35_-N_2_), necessarily WC.
- Parameter 3: presence or absence of the strong hydrogen bond between U_33_ 2’OH and N_35_ that only occurs when N_35_ is a purine (R) (13).

Considering the sum **S** of hydrogen bonds defined by the three parameters, it was shown that when **S** ≤ 5, the considered codon belongs to a two-fold degenerate family, while it belongs to a four-fold degenerate family if **S** > 5 (12). The WC geometry of N_35_-N_2_ is critical since it is closely monitored by A1492 and G530 of the decoding center. These residues control 30S closure (3,4) through an induced-fit mechanism which triggers GTP hydrolysis on EF-Tu and the subsequent release of the tRNA for accommodation (14–16). This geometry can be perturbed by non-WC base pairs at the third position of the codons. The model shows that penalizing N_34_-N_3_ mismatches can sufficiently alter that geometry to prevent the decoding center from adopting a productive configuration. With **S** > 5, any perturbation by the four possible base pairs between (usually) U_34_ and N_3_ is contained by N_35_-N_2_, and superwobbling is possible, whereas base pairing is restricted to simple wobbling when **S** ≤ 5, which has allowed the encoding of two different amino acids (or an amino acid and the stop function) by the considered N_1_N_2_ doublet during the expansion of the initial genetic code.

At the time when this model was published, the dynamics of the decoding center was unknown, and its three residues (A1493, A1492 and G530) were assumed to be either all in the OFF or all in the ON state (resp. *syn* and *anti* for G530), the latter case corresponding to a situation where they are tightly packed and form hydrogen bonds along the minor groove of the anticodon–codon complex. In that state, the ribosome is engaged to accept the tRNA (3,4,14,17). This *a priori* type of dynamics implied that an essential aspect of the model was unsatisfactory: in the all-OFF state, the respective contributions of the hydrogen bonds of N_35_-N_2_ and N_36_-N_1_ to the stability of the N_35_-N_2_ base pair were identical (as it reflects in **S**), which was physically implausible. To resolve this inconsistency, it was envisioned (although not clearly stated) that residue A1493 would always be in the ON state when N_36_-N_1_ and N_35_-N_2_ were complementary, even in the occurrence of penalizing mismatch at the third position. In that state, this residue binds to N_36_-N_1_ through a type I A minor interaction, which is stronger with G_36_-C_1_ and C_36_-G_1_ as compared to A_36_-U_1_ and U_36_-A_1_, thereby amplifying the difference already present between these pairs. A structural context with N_36_-N_1_ as a triple base pair (N_36_-N_1_-A_1493_) would explain why N_35_-N_2_ and N_36_-N_1_ had an apparent similar weight in the stability of the N_35_-N_2_ base pair. It implied, however, that the decoding center would be already partially ON even though the tRNA could still be rejected by the ribosome. Insights into the dynamics of the decoding center were necessary to clarify this issue.

Two recent cryo-EM analyses (15,18) were able to highlight details of this dynamics, which allow us now to confirm the anticipated role of A1493 in degeneracy and reveal that the degeneracy of the genetic code results not only from reduced structural constraints at the wobble position but also from the participation of A1493 in the stability of the N_35_-N_2_ base pair. These new results clearly show that the whole organization of the genetic code is intimately connected to the ribosome itself.

In the first part of the paper, an improved structural model of degeneracy is outlined in the light of the cryo-EM analyses of Loveland *et al.* (15) and Fislage *et al.* (18).

The second part relates the dynamics of the decoding center outlined in the first part to the evolution of decoding on the ribosome, an analysis underpinned by models of ribosome evolution (19,20). We show that this dynamics is consistent with an early appearance of A1493 and A1492 on helix h44, at a time when no catalytic site was present and when an early kinetic scheme of translation that did not include tRNA accommodation was prevailing. In this early kinetic scheme, inferred from a physico-chemical correlation in the genetic code, our analysis suggests that the initial role of A1493 and A1492 was to allow a relaxation of base pairing specificity at the third position of the codons through the compensatory strengthening they implemented at the first position, which gave rise to degeneracy. Kinetics considerations suggest that the peptidyl transferase center (PTC) was the next major acquisition by the ribosome, while proofreading (21–23) arose at a later stage together with tRNA accommodation and the initial form of EF-Tu•GTP. Our analysis leads us to conclude that the controlled hydrolysis of EF-Tu’ GTP through 30S closure by induced fit was a latecomer mechanism, implemented when G530 was acquired by the decoding center.

## MATERIALS AND METHODS

### Analysis of ribosome structures

Crystal and cryo-EM structures of ribosomes complexed with tRNAs or tRNA fragments (15,18,24) were retrieved from the *protein databank* website and analyzed with the *Pymol* software.

## RESULTS

### PART I : Degeneracy on modern ribosomes

#### The structural model of degeneracy is consistent with cryo-EM data

Recent cryo-EM investigations on the decoding mechanism of the ribosome have allowed the identification of three different states of the decoding center and the A-site tRNA in the timeline from initial tRNA ribosome binding down to 30S closure (15,18). Following Fislage *et al.*’s notations (18) (see Figure 7 of their publication), these states are: initial tRNA binding, tRNA sampling and engaged state, the latter state corresponding to a closed 30S subunit, in which the ribosome commits to accept a tRNA. Although the temporal order of these states could be questioned (a common issue in cryo-EM), this order is consistent with our own analysis, as outlined below.

The structures show that with a single mismatch at either the first or the second position of the codon, or in the cognate case, residue A1493 moves to and remains in the ‘ON’ position during tRNA sampling, i.e. flipped out of helix 44 and in N_36_-N_1_ minor groove binding position.

**Figure 2.**
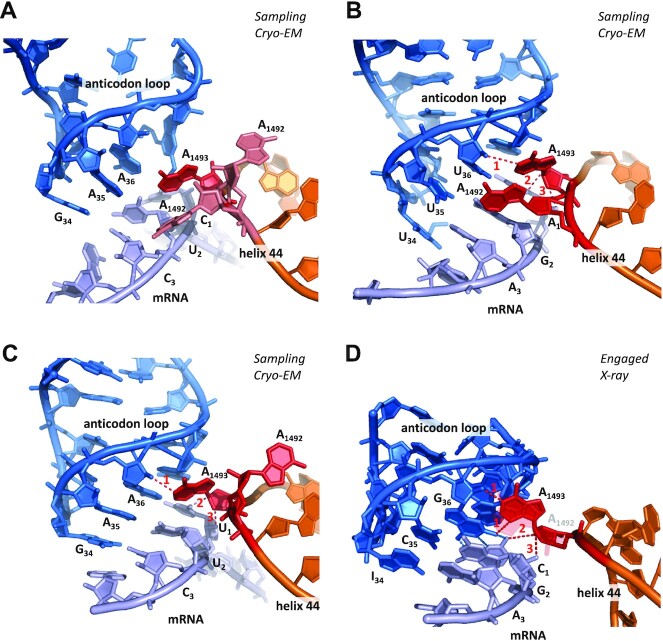
Cryo-EM (**A**–**C**) and X-ray (**D**) structures of anticodon–mRNA complex within the decoding center of the ribosome (for clarity, G530 and helix h18 are not shown). (A) Non-cognate interaction, with AC mismatch at the first position in the state of tRNA sampling (pdb 5wfk, (18)). Although A1493 is ON, no hydrogen bond with the minor groove can occur. CryoEM resolution is 3.4 Å. (B) Non-cognate interaction, with GU mismatch at the second position in the state of tRNA sampling (pdb 5uyp, (15)). A1493 binds to the minor groove. Hydrogen bond D-A lengths are 1: 3.6 Å; 2: 3.0 Å; 3: 4.5 Å (avg.: 3.7 Å). CryoEM resolution is 3.9 Å. (C) Cognate interaction in the state of tRNA sampling (pdb 5uyl, (15)). A1493 binds to the minor groove. Hydrogen bond D-A lengths are 1: 3.0 Å; 2: 3.1 Å; 3: 3.8 Å (avg.: 3.3 Å). CryoEM resolution is 3.6 Å. (D) X-ray structure of a cognate interaction (pdb 1xnq, Murphy and Ramakrishnan 2004) illustrating an A minor interaction with a GC base pair at the first position. Hydrogen bond D-A lengths are 1: 2.6 Å; 1’: 2.9 Å; 2: 3.3 Å; 3: 2.5 Å (avg.: 2.8 Å). Compared to pdb 5uyl, examination of the 5uym pdb structure suggests that the shorter length of these bonds results from A1493 and A1492 being both bound to the anticodon–codon complex. X-ray resolution is 3.05 Å. In order to highlight hydrogen bonds, the angle of view was tilted compared to the other structures, and A1492 is semi-transparent. Overall, A1492 is found about 50% of the time in the ‘ON’ state during tRNA sampling (18). Specific densities of A1492 are such that it is 50% ON/50% OFF in the 5wfk structure (light pink), ON in the 5uyp structure (red) and OFF in the 5uyl structure (red).

With a A_36_-C_1_ mismatch at the first position, A1493 does not form hydrogen bonds with the minor groove, no AC pair being formed (Figure 2A). With a G_35_-U_2_ mismatch at the second position, a A_36_-U_1_ base pair does form, and A1493 binds to its minor groove, although none of its three hydrogen bonds is optimal (Figure 2B). In the cognate case, A1493 binds to the minor groove and forms h-bonds during tRNA sampling (Figure 2C).

There is no existing structure with a forbidden base pair at the third position only, for which the model predicts that A1493 would, likewise, bind to the first base pair during tRNA sampling. The above data, however, clearly support this possibility.

Because the wobble position is two base pairs away from the A1493 binding site, a N_34_-N_3_ mismatch generates a smaller perturbation at the A1493 binding site than a N_35_-N_2_ mismatch, for which A1493 A minor binding during tRNA sampling is now confirmed (Figure 2B). A complete demonstration would, however, require a structure with a base pair more penalizing than G_34_-U_3_ at the third position, e.g. U_34_-U_3_ or U_34_-C_3_ (U_34_ is almost always involved in superwobbling, see (7,9)).

In brief, cryo-EM analyses have revealed that the A1493 residue of the decoding center binds to the minor groove of N_36_-N_1_ during tRNA sampling if this base pair is Watson–Crick, a binding that *further stabilizes the complex* during the time it is tested by residues A1492 and G530 for 30S closure.

#### Degeneracy in the genetic code is established through a major contribution by A1493

**Figure 3.**
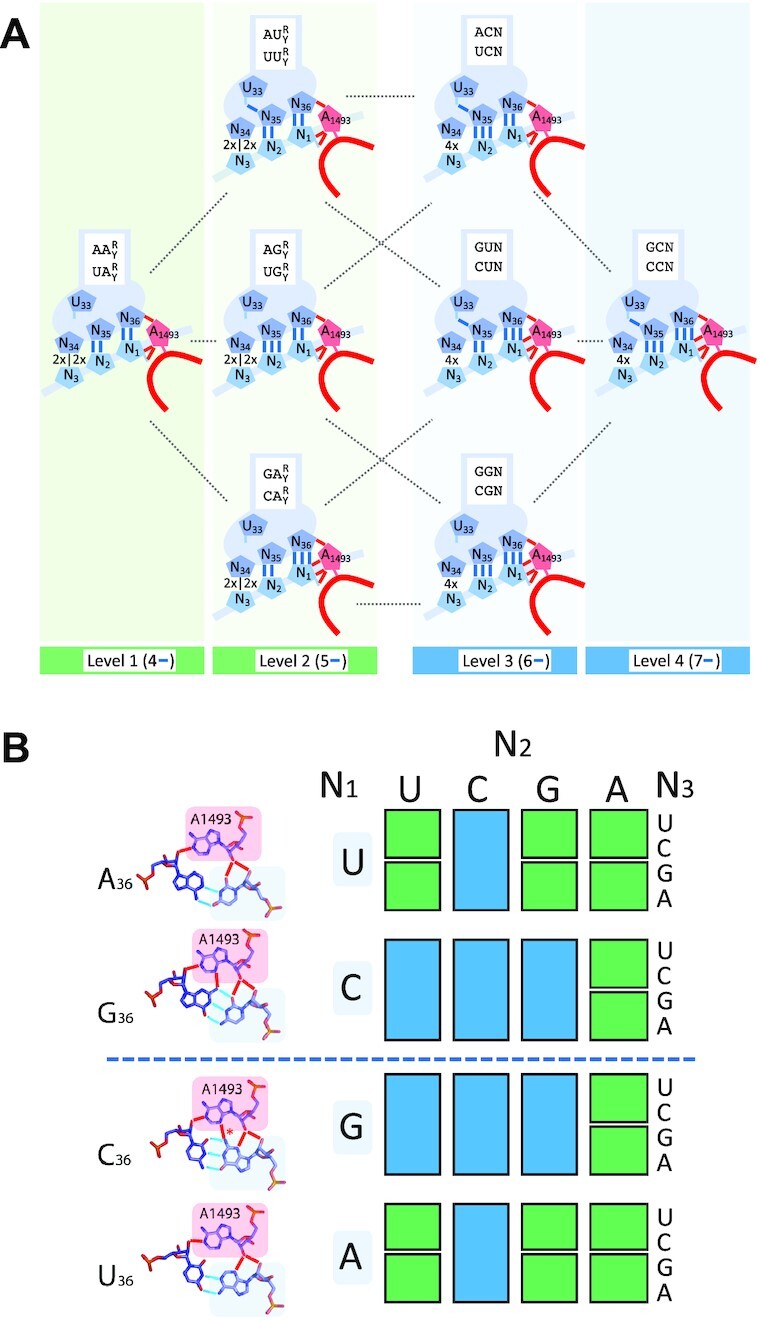
Relation between hydrogen bonding patterns involved in the stability of the WC geometry of N_35_-N_2_ and degeneracy. (**A**) Levels of stability of the WC geometry of the N_35_-N_2_ base pair during tRNA sampling, as determined by hydrogen bonds associated with Lagerkvist’s parameters (in blue) and residue A1493 (in red). Levels 1 and 2 specify contiguous two-fold degenerate codon families (ending with either R or Y, depicted as 2x|2x at the wobble position), while levels 3 and 4 specify four-fold degenerate codon families (ending with N, depicted as 4x at the wobble position). Corresponding codon families are shown in white boxes. R = purine, Y = pyrimidine, N = any of the four bases. (**B**) Yeast or human mitochondrial genetic code table highlighting the two families of degeneracy (same color code as in A). Amino acids are not specified to point out that they are not primarily involved in the determination of these families. The A minor interaction between A1493 and N_36_-N_1_ is shown on the left. All shown hydrogen bonding patterns were found in experimental structures (see Figure 2), except that of C_36_-G_1_-A_1493_, for which no structure could be identified in the pdb database. In that case, the only hypothetical hydrogen bond, highlighted with an asterisk*, is expected to occur similarly as for the G_36_-C_1_-A_1493_ configuration due to the position of the G_1/36_(C_1_-NH_2_) amino group at the center of the base pair.

The cryo-EM data of Loveland *et al.* and Fislage *et al.* (15,18) allow us to refine the structural model of degeneracy previously described (12). Figure 3A highlights the four different levels specifying the stability of the WC geometry of the N_35_-N_2_ base pair during tRNA sampling in the situation when both N_36_-N_1_ and N_35_-N_2_ are complementary. The two lowest levels attribute a two-fold degeneracy to the corresponding codons, while the two highest levels attribute a four-fold degeneracy. As a result of the equivalence of Lagerkvist’s parameters, levels 2 and 3 are degenerate in such a way that three configurations of hydrogen bonding patterns are possible. Remarkably, to each configuration correspond two sets of codons related by A_1_ ↔ U_1_ or G_1_ ↔ C_1_ permutations (indicated on each anticodon stem in Figure 3A). Consequently, when A_1_ (G_1_) and U_1_ (C_1_) are mirror ordered with respect to the center of the table (dashed line), the two degeneracy families are also symmetrically arranged with respect to the center (Figure 3B).

According to the analysis, the most remarkable effect that occurs when both N_36_-N_1_ and N_35_-N_2_ are complementary is the *positive* selection of tRNAs enforced by A1493: the strengthening of N_35_-N_2_ resulting from N_36_-N_1_ A minor binding enables the acceptance of some tRNAs with non-WC base pairs at the third position, whereas tRNAs are counterselected when N_36_-N_1_ and/or N_35_-N_2_ are not complementary (3,4,15,18).

The involvement of A1493 in degeneracy provides an explanation for why Lagerkvist’s parameters are equivalents (Figure 3A): each increase in the level of stability of N_35_-N_2_ occurs upon the addition of either 1 *local* hydrogen bond (U_33_-N_35_ or N_35_-N_2_, in blue) or 2 hydrogen bonds on the *neighboring* triple base pair (N_36_-N_1_-A_1493_, one in blue and one in red), revealing that these two possibilities are equivalent in term of the added stability to N_35_-N_2_.

A striking aspect of the model is that no stacking parameter is required. It suggests that the high number of hydrogen bonds involved (7 to 11) confer structural energies that dominate over the variability of the stacking interaction, which further corroborates the implication of A1493 in degeneracy. The number of these hydrogen bonds is invariant upon A_1_ ↔ U_1_ or G_1_ ↔ C_1_ permutations. In the case of G_36_-C_1_ and C_36_-G_1_, this property stems from the position of the G_1/36_(C_1_-NH_2_) amino group at the center of the base pair (Figures 2D and 3B). While the length of these bonds may vary (and may not be accurately determined due to structure resolution), they can all be categorized as ‘strong’: during tRNA sampling, the average DA distance is 3.3 Å for the hydrogen bonds of the A minor between A1493 and the U_1_-A_36_ base pair and 3.15 Å for the hydrogen bonds involved in U_1_-A_36_ and U_2_-A_35_ base pairing (Figure 2C).

Although stacking is not a parameter, N_37_ stabilizes the N_36_-N_1_ base pair by stacking on it, an effect that is optimal since this base is a conserved purine (13). Stabilization is further enhanced when N_37_ is modified (25–29), and the extent of modification negatively correlates with the G + C composition of the anticodon (25,28), indicating that this base also contributes to an adjustment of the overall stability of each anticodon–codon interaction, and is thus possibly a hidden requirement to the observed degeneracy. Deformation of the tRNA body has also been shown to affect the extent of wobbling at the third position (see Summary and Discussion section).

With regard to the present analysis, the *directional* nature of hydrogen bonds, which is more restrictive compared to that of stacking, and is thus is more prone to keep residues in a given orientation, plausibly explains why they play a predominant role in the stability of the *geometry* of the N_35_-N_2_ WC base pair, which is the decisive criteria for ribosome closure (15,18). In a situation relevant to degeneracy (Figure 3A), this geometry is preserved if the network of hydrogen bonds stabilizing N_35_-N_2_ is strong enough to contain the perturbation generated by a given non-canonical N_34_-N_3_ base pair.

### PART II : Evolution of decoding on the ribosome

#### The induced-fit mechanism is a late acquisition of the decoding center

**Figure 4.**
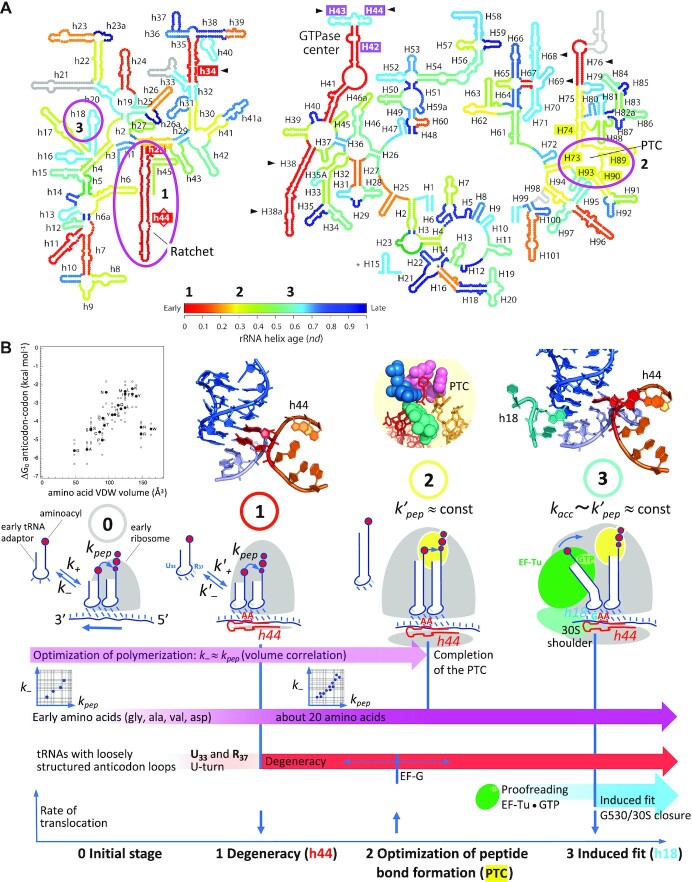
Evolution of rRNA structures in the model of Harish and Caetano-Anollés and evolution of decoding in translation based on the analysis of degeneracy. (**A**) rRNA evolution. Three specific helices (or groups of helices) involved in transitions in the evolutionary model of decoding are highlighted. Adapted from Harish and Caetano-Anollés (19). (**B**) Evolutionary model of decoding on the ribosome. From the origin until the advent of the PTC, a Michaelis–Menten type of kinetic inferred from the volume correlation (11) governs the rate of translation, with tRNA association (***k_+_***) and dissociation (***k-***) rate constants, and a kinetic constant of peptide bond formation (***k*_pep_**), sometimes called ***k*_cat_** in earlier works (11,31,32). The advent of U_33_ and R_37_, as well as helix h44 (A1493 and A1492) modulated these kinetic constants (***k'_+_***, ***k'-***). The step of tRNA accommodation, that appeared concomitantly with EF-Tu, is characterized by an approximately uniform kinetic constant ***k*_acc_**. Relevant features are shown above each evolutionary stage: (0) Volume correlation. The anticodon–codon Δ*G*_0_s are assumed to relate to dissociation rate constants ***k-***s through ***k-*** ∼ exp (Δ*G*_0_) (11), while data on intramolecular reactions suggest a similar exponential dependence between ***k*_pep_** and van der Waals volume (33,34), with some apparent exceptions (asn, arg, trp). (1) Decoding center with h44 only. (2) Peptidyl transferase center (PTC). (3) Whole decoding center with helix h18. See text for additional explanations. Note that all three considered transitions highlighted in A and B concur, although these two models were established essentially independently.

The implication of A1493 in degeneracy shows that the implementation of unspecific pairing at the third position of the codons arose at the time when the ribosome acquired residue A1493 on helix h44. Remarkably, two analyses suggest that the segment of h44 where A1493 and A1492 are located appeared early in the evolution of the ribosome, whereas helix h18, harboring G530, emerged at a much later stage (19,20) (Figure 4A). The latter residue has a major role in the induced-fit mechanism: it drives 30S closure (15,18), which triggers GTP hydrolysis on EF-Tu, thereby releasing the incoming tRNA for accommodation (14,16,30). The mentioned models on ribosome evolution are thus consistent with the induced fit of the decoding center being, logically, established later than degeneracy.

The connection between the successive emergence of helices h44 and h18 and these fundamental aspects of translation must be underscored. The mechanism itself reflects this evolutionary succession: A1493 *first* binds to the minor groove, while A1492 fluctuates between ON and OFF states; *only then* can A1492 and G530 fully bind to the complex in the cognate case, thereby achieving ribosome closure (15,18).

#### A model of the early translation before the appearance of helix h44

The involvement of A1493 in degeneracy highlighted by the present analysis, and the coherence of the sequential buildup of the decoding center in the evolutionary models of Harish and Caetano-Anollés (19) and Petrov *et al.* (20) motivated us to outline a model of evolution of ribosomal decoding based on the identified role of A1493 and a plausible form of the earliest kinetic scheme of translation (32).

This kinetic scheme (Figure 4B, left) was established from an interpretation of a physico-chemical correlation in the genetic code called the volume correlation (11,31,35). This correlation suggests that at the origin of translation, the lifetime of the association between a tRNA and a complementary codon was about equal to the characteristic time required by the aminoacyl carried by this tRNA to make a peptide bond, which was strongly side-chain dependent. This adjustment, which can be expressed with kinetic constants as ***k-***_anticodon-codon_ ≈ ***k*_pep_**_aminoacyl_ (in short: ***k-*** ≈ ***k*_pep_**), implies that the aminoacyls were in immediate position for forming a peptide bond upon tRNA codon binding—i.e. there was no tRNA accommodation at the origin—while not being confined inside a catalytic site, which would have standardized the ***k***_pep_s to an approximately uniform value, an action that is achieved by the peptidyl transferase center (PTC) of modern ribosomes (35). An elementary Michaelis–Menten kinetic scheme comprising the above kinetic constants best encapsulates these features (Figure 4B, left).

In this kinetic scheme, the dissociation rate constant of the tRNA on the P site after peptide bond formation is naturally assumed to be equal to the ***k-*** on the A site, a symmetry that explains why, in the early kinetic scheme, the rate of translation is optimal precisely when ***k-*** ≈ ***k_pep_*** occurs for all tRNA:aminoacyl couples (Cibils *et al.*, in prep.). Key aspects that account for this property are briefly described below.

In order to ensure peptide bond formation, it seems the stronger the anticodon–codon interaction the better: hypothetical anticodons made up of a large number of nucleotides (low ***k-***) would make a tRNA stay a long time on a cognate codon (much longer than the time required by the reaction) and peptide bond formation would always occur. However, once peptide bond formation has occurred, a high rate of translation requires that the (previous) tRNA dissociates as fast as possible (high ***k-***) so that translocation can take place to let the subsequent codon available for a next incoming tRNA.

On the whole, there is an optimum in the lifetime of the anticodon–codon association that ensures (i) a reasonably good probability of peptide bond formation *p* = ***k*_pep_** / (***k-*** + ***k*_pep_**), essentially half of the time according to the above equality, and (ii) a fast dissociation of the tRNA that has done its job and must leave the complex. The volume correlation suggests that most tRNA:aminoacyl couples follow the ***k-*** ≈ ***k*_pep_** equality enabling a high translation rate, which also provides a rational for the size of three nucleotides the (anti)codons.

Another line of analysis suggests that the volume correlation may, alternatively, reflect an optimization in decoding fidelity. In this regime, ***k-*** is slightly smaller than ***k*_pep_**, which ensures the best discrimination of complementary interactions from noncomplementary ones, a property that directly results from the theory of optimal decoding developed by Y. Savir and T. Tlusty (36,37). The volume correlation could either reflect an optimization in decoding or in the rate of translation, or a solution in between these two regimes, that are very close.

#### The appearance of helix 44 with A1493 and A1492 generated a decoding transition on the ribosome

In the context defined by the model of the early translation outlined in the previous section, a straightforward consequence of the strengthening of the N_36_-N_1_ base pair that occurred when A1493 became functional on h44 was a relaxation of base pairing specificity at the third position of the codons, a rebalancing scheme that would have overall preserved the ***k-***, and thus the rate of translation. In a context of a limited variety of tRNAs, this action of A1493 presumably led to an increase in the processivity and accuracy of translation, discussed below.

Because a mismatch perturbs the geometry and stability of neighboring base pairs along a double helix, the type I A minor binding achieved by A1493 could have been optimal only at the first position, i.e. two base pairs away from the third position (where tolerated mismatches would occur), which may explain why this solution was selected.

Although models of ribosome evolution may not predict whether A1493 and A1492 were both initially present on h44 (19,20), this possibility is plausible since the dynamics of A1493 would likely be altered without A1492, and the type II A minor binding achieved by A1492 (3), which is more tolerant to mismatch (it does not bridge over N_35_-N_2_), may contribute to N_36_-N_1_ stabilization. This binding occurs ∼50% of the time during tRNA sampling (18). In that state, the tRNA is partially bent, a feature associated with presence of EF-Tu that allows an optimal substrate selection through deformation (17,36–39). Because EF-Tu, an elaborate protein cofactor, could not have occurred at the origin of translation (which is consistent with an absence of tRNA accommodation, inferred from the volume correlation), it can be affirmed that the stem of initial tRNA adapters did not undergo such deformation. In that case, A1492 would bind 100% of the time to the complex upon tRNA codon association, similarly as it does with fully accommodated tRNAs on modern ribosomes. A fully bound A1492 may contribute to an optimal strengthening mediated by A1493 in the situation when both N_36_-N_1_ and N_35_-N_2_ are Watson–Crick.

**Figure 5.**
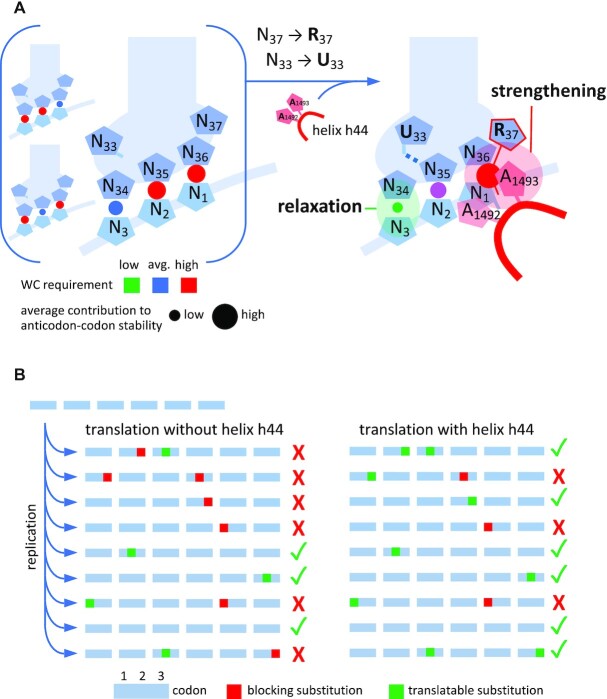
Evolutionary transition 1: from early tRNA anticodon loops and no decoding center to U_33_ and R_37_ -shaped anticodon loops and helix h44 on the ribosome (A1493 and A1492). (**A**) Left: initial loop of tRNA adapter, with little structuration, bound to a codon in an absence of decoding center. Although the shape of the loop might provide a high flexibility to the base pair at the third position, single GU wobble base pairs may also occur in pos. 2 or 1 (background) while still providing enough stability to ensure peptide bond formation in the early translation mechanism. Right: the advent of R_37_ and helix h44 strengthened the anticodon–codon interaction at the first position, while the U-turn (U_33_) helped relax base pairing specificity at the third position. R_37_ stacking on N_36_-N_1_ is schematized with a thin red line. (**B**) Translation of early coding sequences: suggested improved processivity resulting from transition 1. Because the early replication mechanism is inaccurate, RNA sequences accumulate mutations, and thus may not always be fully translated due to reduced sets of tRNAs (left). The advent of h44 together with anticodon loop structuration (see A) provided an improved processivity during translation by lowering base pairing requirement at the third position (right).

Structural and functional considerations suggest that both the processivity and accuracy of translation increased when residues A1493 and A1492 became functional on helix h44:

Processivity of translation: In the proposed Michaelis–Menten kinetic scheme of the initial translation (Figure 4B), the relaxation in base pairing specificity that occurred at the third position of the codons through the action of A1493 and A1492 may have allowed a given set of different transfer tRNAs, necessarily limited at the origin, to be more tolerant to mutations incorporated at the third position during replication (Figure 5), and thus translate longer sequences.

As there was initially no strong geometrical requirement for the base pair at the second position in the absence of G530 and induced-fit mechanism, unspecific base pairing at the third position, that perturb the N_35_-N_2_ geometry, was plausibly less stringent than that occurring on modern ribosomes.

**Figure 6.**
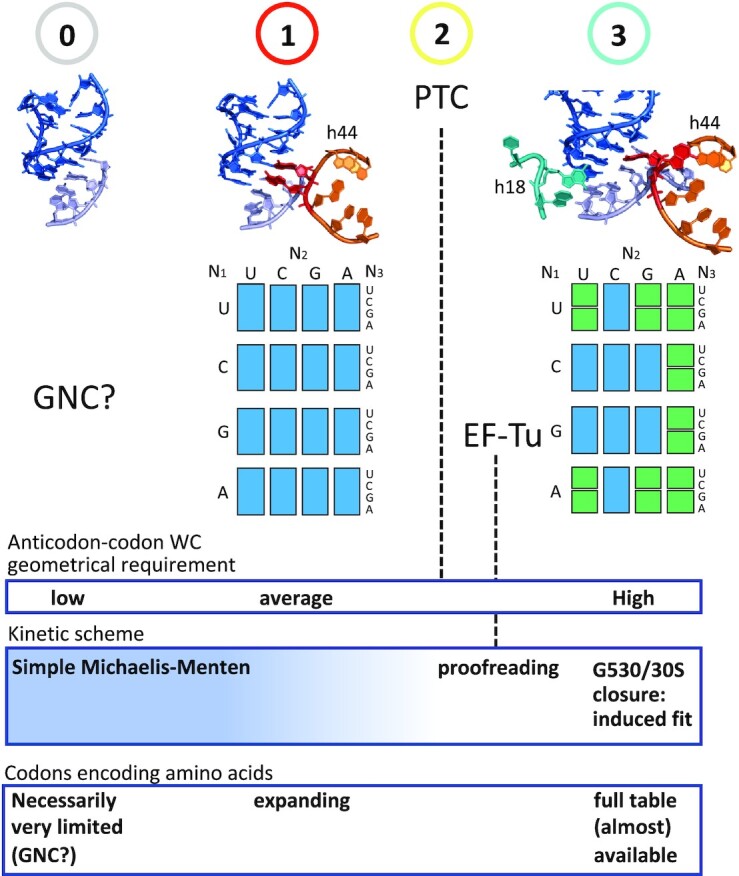
Anticodon–codon interaction and codon degeneracy in the genetic code during ribosome evolution. From an early hypothetical structure with no decoding center (initial state, 0), in which the properties of a GNC code may have provided a required stability to an early translation system (42,44), evolutionary models and the dynamics of the decoding center suggest that helix h44 with A1493 and A1492 appeared first (transition 1), which enabled an extended degeneracy at the third position (blue boxes). The completion of the PTC (transition 2) and the appearance of EF-Tu (proofreading) necessarily occurred before a controlled hydrolysis on EF-Tu by the decoding center through G530 and 30S closure (transition 3), which gave rise to modern degeneracy. Inferred kinetic scheme and codons occurring from stage 0 to transition 3 are indicated at the bottom.

A1493 and A1492 binding would compensate for the loss in anticodon–codon stability generated by mismatches at the third position within a simple rebalancing scheme (Figure 5A, Table 1). It suggests to us that the four codons belonging to any of the 16 N_1_N_2_ doublets of the genetic code may have been translated by a single tRNA upon the action of h44 in an all four-fold-degeneracy regime following transition 1 (Figure 6, center). This possibility does naturally not imply that all 16 doublets were encoding amino acids, at least immediately following this early transition. The acquisition of R_37_ and U_33_ on the anticodon loop, that presumably also occurred early in the evolution of the tRNAs, respectively reinforced the N_36_-N_1_ base pair through R_37_ stacking and provided an extended conformational freedom to N_34_ at the edge of the U-turn (40), in an apparent synergism with the effect of h44 (Figure 5A). The early replication mechanism being inaccurate, the arising degeneracy most likely improved the processivity of translation among mutated copies of early RNA genes (Figure 5B).

**Table 1.** Evolutionary decoding transition 1: appearance of A1493 and A1492 on h44

Expected effect of **A1493 and A1492** (the arrow stands for the evolutionary transition)	Comment
**Cognate interaction:** ***k* *_-_*** $\underset{\longrightarrow}{^{A1493\& A1492}}$ ***k* *’* *_-_*** < ***k_-_***	Higher probability of peptide bond formation
**Mismatch at first position:** ***k-* *_MM1_*** $\underset{\longrightarrow}{^{A1493\& A1492}}$ ***k-* *_MM1_***	A1493 cannot bind. Low probability of peptide bond formation
**Mismatch at second position:** ***k-* *_MM2_*** $\underset{\longrightarrow}{^{A1493\& A1492}}$ ***k* *’-* *_MM2_* *<* *k-* *_MM2_***	A1493 could not optimally bind. Unclear
**Mismatch at third position:** ***k-* *_MM3_*** $\underset{\longrightarrow}{^{A1493\& A1492}}$ ∼ ***k-_cognate_***	A1493 optimally binds. Mismatch accepted at third position. MM3 - A1493 and A1492 compensation: basis of degeneracy

Because wobble base pairing could occur at any codon position and still lead to peptide bond formation before the advent of h44 (Figure 5A, left), one should wonder how extensive miscoding could be overcome at the origin. As already suggested by F. Crick, the most plausible solution that occurred was a minimal set of anticodons and codons (41). Several lines of evidence suggest that the ‘GNC’ code (where N is A, G, C or U) was prevailing at the origin of translation, which would have overcome miscoding while simultaneously managing frameshifting and frame indeterminacy at that stage (42–44) (Figure 6, left).

Accuracy of translation: a GU wobble base pair is only slightly less stable than an AU base pair (45), but it is *tilted* compared to a regular WC base pair. In the context of the N_36_-N_1_-A_1493_ triple base pair, no such degree of freedom is available due to *planar* constraints: in order for A1493 to establish optimal hydrogen bonds with N_36_-N_1_, this base pair has to display a WC geometry (3,4) (Table 1).

Thus, an increase in the dimensionality of the complex at the first position, which reduces the conformal freedom of the N_36_-N_1_ base pair, is associated with an increased selectivity. Although extensive investigations will be required to establish how A1493 and A1492 improve the fidelity of translation without G530 and the associated induced-fit mechanism, a prevalence of mismatches at the third position should increase the overall accuracy of decoding through a higher fidelity in positions 1 and 2, required to keep the ***k-*** below a critical value enabling peptide bond formation.

It should be mentioned that Satpati *et al.* (46) found out through molecular dynamic simulations that mismatches on modern ribosomes are penalized essentially as a result of water exclusion due to the binding of A1493, A1492 and G530: missing hydrogen bonds occurring in mismatches cannot be compensated through hydrogen bonding with water. This effect could already partially occur without loop h18 and G530.

Another effect resulting from the action of h44 must be considered: because the A-site tRNA and the RNA template became caught by A1493 and A1492 upon anticodon binding, the rate of translocation necessarily slowed down (Figure 4B, bottom). On modern ribosomes, the grip of the decoding center constitutes a barrier to translocation, which is overcome by the elongation factor EF-G and the free-energy available from the hydrolysis of a GTP (47–50). Without helix h18 and G530, translocation could still spontaneously occur through thermal fluctuations (51). A consistent evolutionary scenario is that an ancestor of EF-G came into the picture after the emergence of A1493 and A1492, which would alleviate the early grip, and make the second transition to G530 and proofreading possible by preventing a catastrophic slowdown of translocation upon building of the full decoding center (Figure 4B, bottom). During evolution, an early fixation of R_37_, which makes an interstrand stacking and thus helps maintain the reading frame, would also best ensure the maintenance of that frame upon appearance of A1493 and A1492 (Figures 4B and 5A). The subsequent appearance of EF-G and R_37_ modifications would further reduce frameshifting events during translocation (26,27,29,50,52–54).

#### Co-evolution of the translation machinery and the genetic code

This section summarizes and brings further justifications to the evolutionary model depicted in Figure 4B. Remarkably, all three major transitions highlighted in this scenario agree with the model of ribosome evolution proposed by Harish and Caetano-Anollés (19) (Figure 4A). While still being consistent with the model of Petrov *et al.* (20), our analysis does not support a very early appearance of the PTC on the ribosome, as suggested by this study (see summary and discussion Section).

*Initial stage (0):* although no strong evidence so far explains the origin of RNA and how the initial translation came about, the volume correlation in the genetic code (11) suggests that the early translation was driven by a simple Michaelis–Menten kinetic scheme (31,32,35). The fixation of U_33_ and R_37_ on the anticodon loops, which improved anticodon–codon associations and helped maintain the reading frame (26,29), was plausibly an early acquisition on all tRNAs.

*First major transition (1):* residues A1493 and A1492 appeared on helix h44. Together with U_33_ and R_37_, they established the basis of modern degeneracy (Figures 5 and 6, center).

*Second major transition (2):* build-up of the PTC. Because this catalytic site confines the aminoacyls in a desolvated environment, the amino groups are more reactive (55). Furthermore, an induced-fit mechanism orients the aminoacyls for nucleophilic attack, which cancels the conformational freedom available to the amino group in solution, that is side-chain dependent (35). As a consequence, all ***k_pep_***s are levelled up to an approximately uniform ***k′*_pep_**value. Thus, at the time of the completion of the PTC, the optimization in rate/decoding that had guided the establishment of the code became obsolete. Free from this constraint, the genetic code could evolve on its own, although codon reassignment is known to have occurred at an extremely low rate—otherwise, the volume correlation would have disappeared.

Because it would break the initial simple MM kinetic scheme (Figure 4B, left), the EF-Tu cofactor could come into the picture only after the optimization of the ***k'_pep_*** achieved by the PTC. In the absence of G530 and induced-fit mechanism, an elementary form of proofreading would occur: most plausibly, GTP hydrolysis on EF-Tu, that leads to the release of the tRNA for accommodation (30), was initially triggered by the docking of the tRNA•EF-Tu•GTP ternary complex onto the saricin-ricin loop of the ribosome, which would occur without requiring the kick by the 30S, thus following the simple clockwork mechanism envisioned by Ninio and Hopfield (21–23), which is independent of the decoding center.

*Third major transition (3):* appearance of helix h18 and the associated induced-fit mechanism (56), that involves G530 anticodon–codon latching and 30S closure (3,4,14,15,18). This large-scale rearrangement docks EF-Tu on the saricin-ricin loop, which triggers GTP hydrolysis (15,16). From the early simple proofreading mechanism (see above), a plausible evolutionary transition was a change in the structure of the ribosome architecture that kept the tRNA•EF-Tu•GTP ternary complex away from the saricin-ricin loop, thus requiring 30S closure to dock the complex.

Available data (55,57) suggest that the kinetic constant of accommodation (***k_acc_***) is of the same order of magnitude as ***k'_pep_*** at physiological pH on modern ribosomes (Figure 4B, right), although this point still needs to be established experimentally. Because of its sensitivity, that is tuned by tRNA deformation (17,38), the induced fit would allow a much sharper discrimination between cognate and near-cognate tRNA through optimal decoding (36,37), thus giving rise to modern degeneracy (Figure 6, right). Base modifications, which could only occur at a late stage with modifying enzymes, will still be required to shape some tRNA anticodon loops so that they can be accepted by the decoding center (58,59), best prevent leaking wobbling between contiguous two-fold degenerate codon families, and ensure reading frame maintenance during translocation.

According to this evolutionary model, the key roles of A1493 and A1492 in degeneracy—i.e. when N_36_-N_1_ and N_35_-N_2_ are both WC—were ‘shifted’ from the step just prior to peptide bond formation (at the origin) to the kinetic step of GTPase activation on EF-Tu (on modern ribosomes). At the origin, A1493 and A1492 binding compensated mismatches at the third position by keeping ***k-*** under a critical value enabling peptide bond formation. In the kinetic step of GTPase activation on modern ribosomes, A1493 still has a compensatory role for unspecific N_34_-N_3_ base pairs through N_35_-N_2_ stabilization on *bent* tRNAs, while A1492 and G530 constitute a switch monitoring the WC geometry of N_35_-N_2_. Because of the sensitivity of the switch, half of the 16 N_1_N_2_ codon doublets, for which the stability of N_35_-N_2_ is near the threshold for A1492 and G530 binding, can be differentiated into two neighboring two-fold degeneracy families. The higher stability of N_35_-N_2_ of the 8 other N_1_N_2_ codon doublets keeps them away from this threshold with any U_34_-N_3_ base pair, implying that these doublets are all four-fold degenerate (see Figure 3).

## SUMMARY AND DISCUSSION

Recent cryo-EM structures have revealed the dynamics of the decoding center of the ribosome during tRNA selection (15,18). Based on these results, the present work shows that residue A1493 of the decoding center plays a key role in degeneracy by strenghtening the N_36_-N_1_ base pair during tRNA sampling, which allows non-specific N_34_-N_3_ pairings to be accepted by the ribosome. This possibility was suspected at the time of an earlier work on degeneracy (12), although it remained unclear because the dynamics of the decoding center was unknown. We now conclude that degeneracy in the modern genetic code is established by a complex comprising the anticodon, the codon and A1493, while a clear-cut distinction between contiguous two-fold degenerate families requires the induced fit mediated by the whole decoding center and modifications on tRNA anticodon loops.

It must be emphasized that degeneracy corresponds to a maximization of wobbling (12), which requires specific tRNAs. Decoding in mitochondria suggests that a uridine in position 34 can almost always achieve superwobbling in four-fold degenerate families (6,7,9), while some uridine modifications, such as uridine 5-oxyacetic acid, are known to further enhance this property (60,61).

However, most bacteria and higher order organisms use more than one tRNA to translate all codons in four-fold degenerate codon families. Although a general structural strategy that prevents superwobbling has not been identified, the absence of this flexibility results from constraints applied to the anticodon–codon interaction, which could occur either through U_34_ modifications, by the choice of the anticodon 32-38 closing base pairs (62) or through the flexibility of the whole tRNA (39).

Furthermore, in twofold degenerate codon families, U_34_ modifications (e.g. xm^5^s^2^U derivatives) are always present, and are required to prevent ‘leaking’ wobbling between families sharing identical nucleotides in positions 1 and 2 (63,64). Codon assignment was, therefore, partially ambiguous before the appearance of modifying enzymes (and still is to some extent). The advent of inosine might explain why AUR and AUY two-fold degenerate families further reorganized into AUG and AU/U,C,A codon boxes. More generally, the extent of wobbling—and thus, degeneracy—is controlled by structural deformations required for the anticodon to achieve proper codon binding in the context specified by the ribosome and EF-Tu (17,36–39), which often requires base modifications (58,59,65).

The reason why degeneracy has remained as the full extension of wobbling is likely that the tRNAs of the earliest simple organisms (perhaps LUCA) pushed the limits of wobbling to the maximum. Once these rules were established and encoded in genomes, it became (almost) impossible to change them although more tRNAs following simpler (or no) wobbling rules were added afterwards, which offered more flexibility and efficiency in decoding.

According to the present analysis, a maximum of 24 degeneracy families may occur—neglecting a few singularities such as that of AUG and AU/U,C,A codon boxes in the canonical genetic code, implying that 23 amino acids may be encoded if only one degeneracy family is assigned to stop codons. In view of the interest in genetic code expansion technology (66–68), one should wonder if there are physical limits to the maximum number of amino acids that may be unambiguously translated (setting aside the issue of aminoacylation). It should first be acknowledged that >30 different tRNAs are still required in *Escherichia coli* for the translation of all its coding genes because superwobbling does not occur in this bacterial species, although tRNAs with inosine in position 34 can translate U/C/A-ending codons, while other modifications such as 5-hydroxyuridine are known to confer additional wobbling capabilities. It implies that it should in principle be possible to encode >30 amino acids with the existing translation machinery. It is still very hypothetical since no existing organism could tolerate massive codon reassignment.

The structural analysis of degeneracy suggests yet another theoretical possibility: if instead of G and C, another set of natural or synthetic nucleotides orthogonal to A and U and pairing with only two hydrogen bonds is used, only two-fold degenerate codon families might occur in the entire table as a result of the low stability of all N_35_-N_2_ base pair. This would allow the unambiguous encoding of 31 amino acids plus one degeneracy family assigned to stop codons.

Although it is unclear whether a set of bases pairing with two hydrogen bonds could have occur at the origin of Life in addition to A and U, no ***k-*** ≈ ***k_pep_*** optimization is possible with only weak base pairs, which may contribute to explain why this scenario did not occur.

While the present analysis shows that hydrogen bonds determine the extent of degeneracy in the genetic code, experiments and molecular dynamic simulations suggest that steric complementarity between the decoding center and the anticodon–codon complex is more important than hydrogen bonds in the selection of cognate tRNAs (69,70). There is, however, no fundamental contradiction between these two results: the network of hydrogen bonds involved in degeneracy contributes to the stabilization of the WC geometry at the second position, which is critical only when a non-canonical base pair occurs at the third position. The expected effect of missing hydrogen bonds is only a reduction of the extent of wobble base pairs accepted by the ribosome: in particular, superwobbling with U_34_ that normally occur would be prohibited when specific hydrogen bonds are missing.

In the evolutionary scenario depicted in Figure 4B, the PTC emerges after helix h44 and before helix h18, in agreement with the analysis of Harish and Caetano-Anollés (19) (Figure 4A). This succession can be justified by the following: because the PTC levelled the kinetic constants of peptide bond formation up to similar ***k'_pep_*** values, it cancelled the ***k-*** ≈ ***k_pep_*** adjustment that had shaped the code from the origin (11,31,35). This early optimization, the trace of which is the volume correlation (11), could not have occurred if the PTC was already present at the origin of translation. According to the present work, the possibility of the A1493(h44)/degeneracy rebalancing is based on this optimization, implying that h44 necessarily emerged before the PTC. Our results thus do not support an early emergence of this catalytic site, as the model of Petrov *et al.* (20) suggests. Another justification of the proposed evolutionary scheme relates to tRNA accommodation, which is part of the proofreading mechanism, and implies the presence of the PTC: the tRNA acceptor arm is funnelled by rRNA helices H89 and H90-92, which are both rooted on this catalytic site (71,72). Also, proofreading implies a commitment of the ribosome to peptide bond formation once the 3’ end of an aminoacyl-tRNA reaches the peptidyl-tRNA, which implies high ***k'_pep_***s of similar values, thus the PTC. We conclude that in the timeline of evolution, the completion of the PTC occurred after the appearance of degeneracy (residues A1493 and A1492) and before EF-Tu/proofreading, the induced-fit mechanism (30S closure) controlled by G530 being necessarily a latecomer.

One of the most striking structural aspect of the decoding center is that its three nucleotides are distributed on two different helices far apart from each other, implying that their simultaneous appearance in the course of the early evolution of the ribosome is highly unlikely. In agreement with evolutionary models (19,20), and with the dynamics of the decoding center (15,18), the major conclusion of the present analysis is that degeneracy arose when helix h44 together with residues A1493 and A1492 appeared at an early stage of the evolution of the ribosome.
